# Optimising older People’s Transition from acute care Into residential aged care through Multidisciplinary Assessment and Liaison (OPTIMAL): protocol for a stepped wedge cluster randomised controlled trial with embedded process evaluation

**DOI:** 10.1186/s12877-025-06187-y

**Published:** 2025-07-28

**Authors:** Rangika L. Fernando, Jonathan Karnon, Maria Crotty, Maria C. Inacio, Madeleine Baillie, Ishita Batta, Alice Bourke, John Forward, Chloe Furst, Robert Jorissen, Georgina Neill, Georgina Szabo, Craig Whitehead, Gillian Harvey

**Affiliations:** 1 Flinders University, College of Medicine and Public Health, Flinders Health and Medical Research Institute, Adelaide, South Australia Australia; 2 Registry of Senior Australians (ROSA) Research Centre, South Australian Health and Medical Research Institute (SAHMRI), Adelaide, South Australia Australia; 3 Southern Adelaide Local Health Network, SA Health, Adelaide, South Australia Australia; 4 Flinders University, College of Nursing and Health Sciences, Caring Futures Institute, Adelaide, South Australia Australia; 5 Central Adelaide Local Health Network, SA Health, Adelaide, South Australia Australia; 6 Northern Adelaide Local Health Network, SA Health, Adelaide, South Australia Australia; 7 Commission on Excellence and Innovation in Health, Government of South Australia, Adelaide, South Australia Australia

**Keywords:** Care transitions, Implementation, Patient readmission, Older adults, Hospital discharge, Residential aged care, Nursing homes, Stepped wedge cluster randomised trial

## Abstract

**Background:**

Care transitions by older adults discharged from hospital require good coordination and communication to prevent adverse events and potentially preventable readmissions. Implementation of multicomponent interventions tailored to patient risk can improve the quality of transitions and reduce potentially preventable readmissions. Several such multicomponent interventions exist, but evidence of their transferability and effectiveness for individuals discharged to residential aged care (RAC) and within the Australian context is required.

**Methods:**

The Optimising older People’s Transition from acute care Into residential aged care through Multidisciplinary Assessment and Liaison (OPTIMAL) trial is a multi-site hybrid type II stepped wedge cluster randomised controlled trial with embedded process and economic evaluations. The study aims to collaboratively implement a bundle of evidence-based interventions with clinicians, administrators, and researchers to provide systematic support for first time discharges to RAC, and to determine the clinical effectiveness, cost effectiveness, and feasibility, acceptability, adoption and fidelity of implementation. The study design is informed by the integrated Promoting Action on Research Implementation in Health Services (i-PARIHS) framework, which will guide implementation and process evaluation. The OPTIMAL intervention will be implemented sequentially over 14 months across selected hospital wards in three local health networks (LHNs) in South Australia. Implementation will occur in a random order, with one LHN transitioning to the intervention phase every four months. It will include all patients 65 years and older discharged from the selected wards to RAC for the first time. Eligible participants will be stratified based on their risk of readmission and receive a customized bundle of interventions relative to their level of risk. Each LHN team will tailor an intervention bundle informed by existing evidence and a registered nurse will facilitate its implementation. The primary outcome is the proportion of participants with emergency department presentations and/or readmissions and/or death within 30 and 90 days of discharge. A total of 1545 participants are expected to be enrolled, commencing 1st September 2024.

**Discussion:**

This study will provide evidence on the transferability and effectiveness of implementing multicomponent and risk stratified enhanced care bundles in the Australian context, and can inform improvement activities for care transitions by older adults.

**Trial registration:**

Australia New Zealand Clinical Trial Registry ACTRN12624001008516, registered 20th August 2024.

**Supplementary Information:**

The online version contains supplementary material available at 10.1186/s12877-025-06187-y.

## Background

The transition from hospital to residential aged care (RAC) can be precarious for older adults. In Australia, approximately 61,000 older individuals enter RAC each year [1], and within the first 90 days of entry almost 23% are reported to have presented to an emergency department (ED) and 18% to have an unplanned hospitalisation[2]. These individuals often have multi-morbidity and complex care needs, making care coordination and continuity essential as they transfer between settings [3]. Breakdowns in care processes during transitions can lead to patients “falling through the cracks”, increasing their risk of medication errors, inappropriate care, and ED presentations or preventable hospital readmissions [3, 4]. Ideal transition care for individuals discharged from hospital bridges this gap to the next provider and setting through several components: early discharge planning; communication of information in a timely, well organised, and accessible manner; medication safety; promoting self-management; enlisting the help of social and community supports; advance care planning; coordinating care across providers and settings; monitoring and managing symptoms after discharge; and timely follow-up after discharge [5].

Interventions to improve the quality of care transitions for older adults address one or more of these components of an ideal transition in care [6–12], and increasing the number of components addressed by an intervention increases the likelihood of its success in reducing readmissions [13]. Depending on when they are implemented, interventions can be classified as pre-discharge (e.g., discharge planning, patient and family education, medication reconciliation, scheduling follow-up appointments); post-discharge (e.g., follow-up visits or phone calls, availability of a hotline, primary care communication plans); or interventions that bridge the transition (e.g., transition coach or navigator, provider continuity, patient-centered discharge instructions) [11]. In-hospital interventions that improve discharge planning and follow-up after discharge, and ensure coordination and continuity of care are shown to be effective in reducing hospital readmissions in older adults [9]. Research into the effectiveness of different transitional care strategies also highlights the need for hospitals to promote patient trust and communicate using plain language [12].

Current evidence on interventions to reduce hospital readmissions in older adults favours the use of multicomponent interventions covering both the pre-and post-discharge periods [9–11]. Interventions targeting patients at higher risk of these events, that have high-intensity and a duration of at least one month are more likely to improve patient outcomes [6, 10]. Stratifying patients based on their risk of readmissions and matching the intensity of the intervention to the individuals’ risk of readmission can also improve the cost-effectiveness of an intervention [14]. Several multicomponent models exist internationally to improve the quality of care during care transitions, including the use of a transitions coach [15], Advance Practice Nurse transitional care model [16–19], the Kaiser-Permanente bundle of interventions [20, 21], projects ReD (Re-engineered Discharge) [22] and BOOST (Better Outcomes by Optimizing Safe Transitions) [23], as well as adaptations of these models [16].

Since interventions to improve the quality of care transitions often consist of several interacting components and are implemented across different settings, health disciplines and organisation levels [24], their success can vary based on the context in which they are implemented (i.e., the internal and external environments and the patient population) and the mechanisms triggered [24]. Interventions act through various mechanisms, i.e., improving the discharge process by simplifying the transition complexity, verifying essential steps in the process, or connecting patients, caregivers and providers; empowering patients through coaching and translating the providers goals to the patient’s goals; and monitoring and anticipating future events to ensure timely and appropriate action [24].

Hospital readmissions are costly, both to the individual and health system [25]. Interventions to improve transition care to and from RAC reduce the likelihood of hospital readmission and significantly decrease length of stay in ED [7]. The large body of evidence on successful models to improve care transitions provides an opportunity to systematically support hospitalised older patients transitioning to RAC, but most evidence is not specific to this group. Individuals discharged from hospital to RAC should be given due focus as RAC residents are older, have more comorbidities and higher levels of physical and cognitive impairment than those living at home [26, 27]. The age at entry to RAC in Australia has increased over the years, along with an increase in frailty, number of comorbidities, and number of prescribed medicines [28]. Individuals being discharged from hospital to RAC often have significant care needs, requiring comprehensive management and care plans that should be communicated to the RAC facility in a timely manner [29]. Individual RAC facilities can vary in their ability to manage complex individuals, based on staffing and availability of equipment and infrastructure, so it is vital that they are equipped with the required information and a single point of contact for queries related to ongoing management of the discharged patient [29].

There is a need for Australian evidence on the transferability and effectiveness of existing models to improve transition care, specifically for individuals discharged to RAC. The Optimising older People’s Transition from acute care Into residential aged care through Multidisciplinary Assessment and Liaison (OPTIMAL) study is designed to bridge this gap, by assessing the transferability and effectiveness of implementing enhanced care bundles to support these individuals in the Australian context. The study aims to collaboratively implement a bundle of evidence-based interventions with clinicians to provide systematic support for older adults being discharged from the hospital to RAC for the first time, and to determine the clinical effectiveness, cost effectiveness, as well as the feasibility, acceptability, adoption and fidelity of implementation.

## Methods

### Trial design

The OPTIMAL trial is a multi-site hybrid type II stepped wedge cluster randomised controlled trial with embedded process and economic evaluation. The hybrid type II design allows simultaneous testing of the clinical effectiveness of the intervention (unplanned hospital readmissions) and feasibility and utility of the implementation strategy [30]. The OPTIMAL study will engage stakeholders involved in, and influencing, implementation to design the intervention and will include a process evaluation to understand the context and process of implementation, any adaptations made, the mechanisms of impact, and their effect on the outcomes [31, 32]. The study design is theoretically informed by the integrated Promoting Action on Research Implementation in Health Services (i-PARIHS) implementation framework, in which implementation success is determined by the facilitation of an innovation (intervention) with the intended recipients in their inner and outer contextual setting [33, 34]. It is recognised best practice to apply a theory-informed approach when designing and testing complex interventions, to provide evidence of the role of context in the study outcomes, and to understand the generalisability of study findings [32, 35]. The i-PARIHS framework will inform the implementation and process evaluation of the OPTIMAL trial.

The implementation strategies used by OPTIMAL, as mapped against the Expert Recommendations for Implementing Change (ERIC) taxonomy, include: adapt and tailor to context, use evaluative and iterative strategies, provide interactive assistance, develop stakeholder interrelationships, train and educate stakeholders, support clinicians, and change infrastructure [36, 37]. The process evaluation will support understanding the causal mechanisms and contextual factors affecting implementation and outcomes [31, 32]. The acceptability, adoption, feasibility, and fidelity of implementing the intervention will be assessed using mixed methods. The methods for the process evaluation of the OPTIMAL trial is described in detail in a separate protocol paper (Fernando RL, Crotty M, Inacio MC, Batta I, Bourke A, Forward J, Furst C, Whitehead C, Shaw S, Shepperd L, Harvey G: Implementing a bundle of interventions to support older adults transitioning from hospital to residential aged care: a protocol for the process evaluation of the OPTIMAL stepped wedge cluster randomised controlled trial, under review).

The trial builds on previous research to develop a tool to predict the risk of hospitalisation and ED presentations among older persons entering RAC for the first time, based on data from South Australia, Victoria and New South Wales (which represents 68% of the Australian cohort of aged care recipients) [2]. This model has been incorporated into a data dashboard that utilises data from the electronic medical record (EMR) to categorise eligible patients into risk categories (low/medium/high) based on their calculated risk of readmission within 90 days of entry into RAC. The dashboard will be visible to clinicians working at the LHN, enabling them to identify eligible patients and to deliver a customised bundle of interventions based on the patient’s level of risk.

### Study setting and eligibility criteria

The trial will be carried out across selected acute and sub-acute care wards in three Local Health Networks (LHN) in South Australia: Flinders Medical Centre, Noarlunga Hospital and Repat Health Precinct in Southern Adelaide Local Health Network (SALHN); Royal Adelaide Hospital and Hampstead Rehabilitation Centre in Central Adelaide Local Health Network (CALHN); and Lyell McEwin Hospital and Modbury Hospital in Northern Adelaide Local Health Network (NALHN). The intervention will be rolled out over 14 months, with interventions starting every four months. Each LHN will be randomised to either the control or intervention phase, with one LHN per randomisation. All selected wards within an LHN will commence the intervention together. In the control phase, LHNs will deliver usual care.

All patients aged 65 years and older who are being discharged from these wards to RAC for the first time will be included. Patients will be excluded if they have a planned surgical intervention which will require readmission within the 90 days following hospital discharge. The intervention will be delivered by the clinical teams in each LHN, which includes geriatricians, rehabilitation physicians, nursing and allied health professionals.

### Intervention

The intervention will be preceded by a two-month establishment phase in each LHN, where all the selected wards will deliver usual care. A modification to the EMR to enable identification of eligible patients will be introduced to clinicians during this phase. This data feeds into a data dashboard (de-identified) to assist risk stratification, which will also be introduced during this phase.

**Fig. 1:**
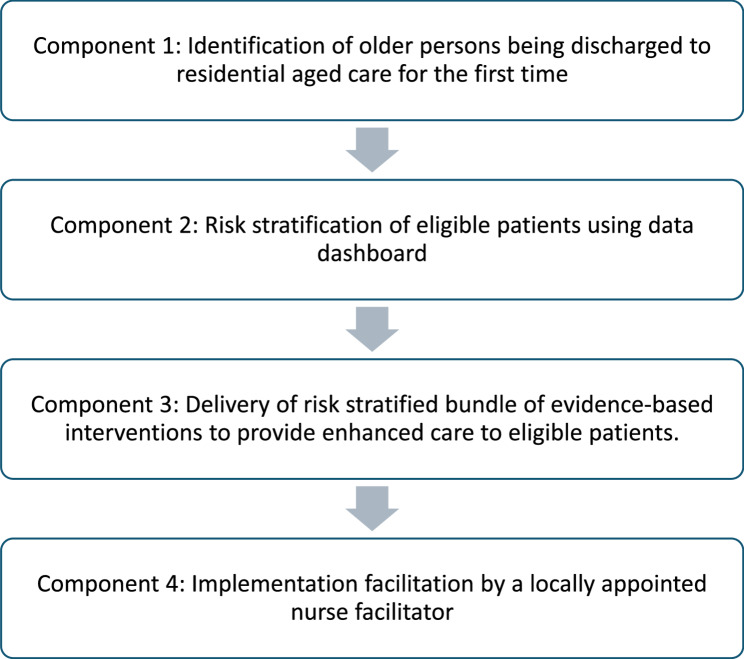
Components of OPTIMAL intervention

The OPTIMAL intervention is a complex intervention and comprises of four core components (Fig. 1). Permissible adaptations in the delivery of each component are defined (supplementary file 1), allowing flexibility to adapt the intervention to suit the LHN context while maintaining the integrity of the four core components [35]. The available evidence will be synthesised and presented and each LHN team will be engaged to design the risk stratified, evidence-based bundle of interventions (Component 3 as shown in Fig. 1) to be implemented at their sites. The choice of interventions can be tailored within each LHN, and may include provision of a standard, same day discharge summary; post-discharge follow-up phone calls; access to a geriatric hotline; medication reconciliation after discharge; and a follow-up visit at the RAC from a registered nurse. A nurse facilitator will be appointed to each LHN at least one month prior to the intervention start to act as an internal facilitator. The LHNs will be responsible to provide any other resources required to implement their chosen interventions.

**Fig. 2:**
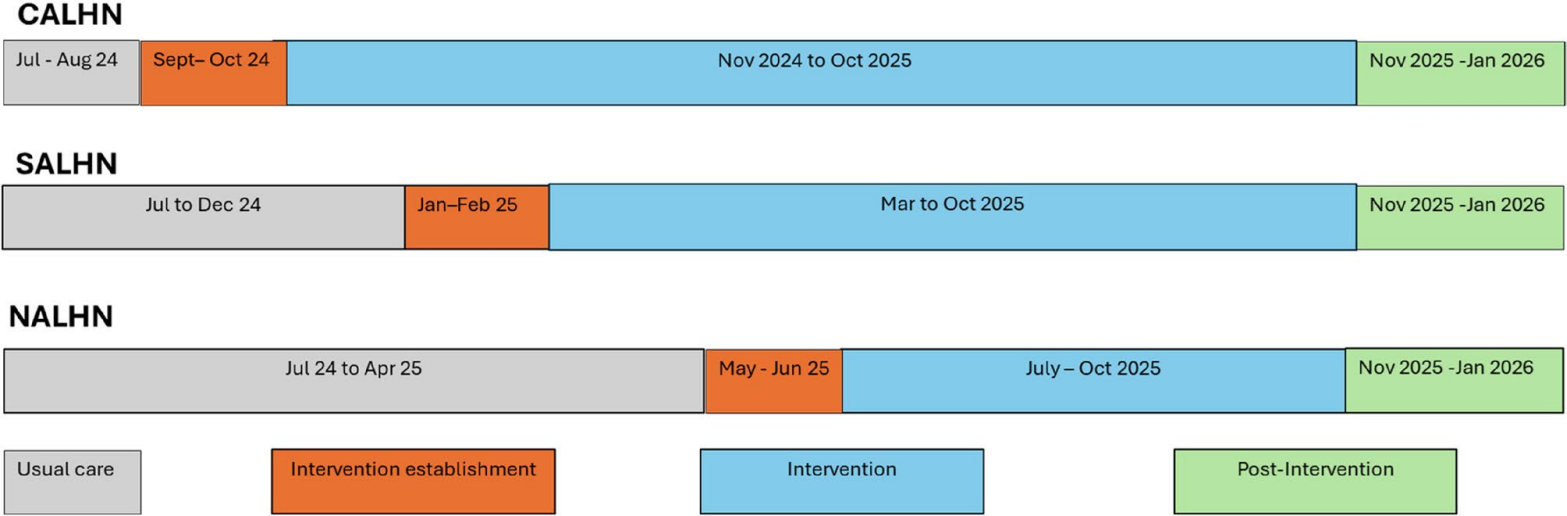
OPTIMAL timeline

In the intervention phase eligible patients will be identified prior to discharge, stratified based on risk of readmission and receive the bundle of interventions relevant to their risk level. The risk of readmission is based on previous research to develop a risk prediction model for hospitalisations for older adults entering RAC using data from the Registry of Senior Australians [2]. This model was adapted for use as a risk profiling tool using variables available in routinely collected EMR data of the participating LHNs and showed good predictive ability (Area under the receiver curve = 0.610 (95% CI 0.5401–0.6458) using SA Health data (from the Department of Health and Wellbeing, Government of South Australia). The data dashboard will highlight eligible patients and their risk category to clinicians working in the LHN. All eligible patients will receive elements of the bundle of interventions based on their assessed level of risk, with those at high risk receiving the earliest and most intensive interventions. For example, high-risk participants may receive medication reconciliation by a pharmacist, follow-up visits and phone calls for a month post-discharge, while those with low risk may only receive a timely discharge summary and a follow-up a phone call. As this is a stepped wedge cluster randomised controlled trial, the length of the intervention phase will be 12 months for LHN 1, 8 months for LHN 2 and 4 months for LHN 3 (Fig. 2). The selected wards will serve as their own controls.

### Outcome measures

The primary outcomes will be the proportions of patients discharged to RAC for the first time who have evidence of presentation to ED and/or readmission and/or death within 30 and 90 days of the date of index discharge, monitored from administrative data.

The secondary outcomes of the study are:

1. Days alive and in RAC within 30- and 90-days following hospital discharge, which will be monitored from administrative data.
2. Proportion of eligible patients receiving the risk stratified bundle of interventions, which will be monitored using data collected from the nurse facilitator.
3. The feasibility, acceptability, adoption and fidelity of implementation, which will be assessed through qualitative interviews with key decision makers in each LHN, a survey questionnaire of participating clinical teams, baseline context mapping and activity logs maintained by the nurse facilitators in each LHN.

### Timeline

Participant enrollment will commence on 1st September 2024, and end on 31st October 2025 (Fig. 2). Delivery of the intervention will begin on 1st November 2024 in LHN 1, 1st March 2025 in LHN 2, and 1st July 2025 in LHN 3. The intervention start will be preceded by a two-month establishment phase in each LHN, during which participants will receive usual care. Enrolled participants will be followed up for at least 90 days after hospital discharge for evidence of outcomes through administrative data.

### Sample size

**Fig. 3:**
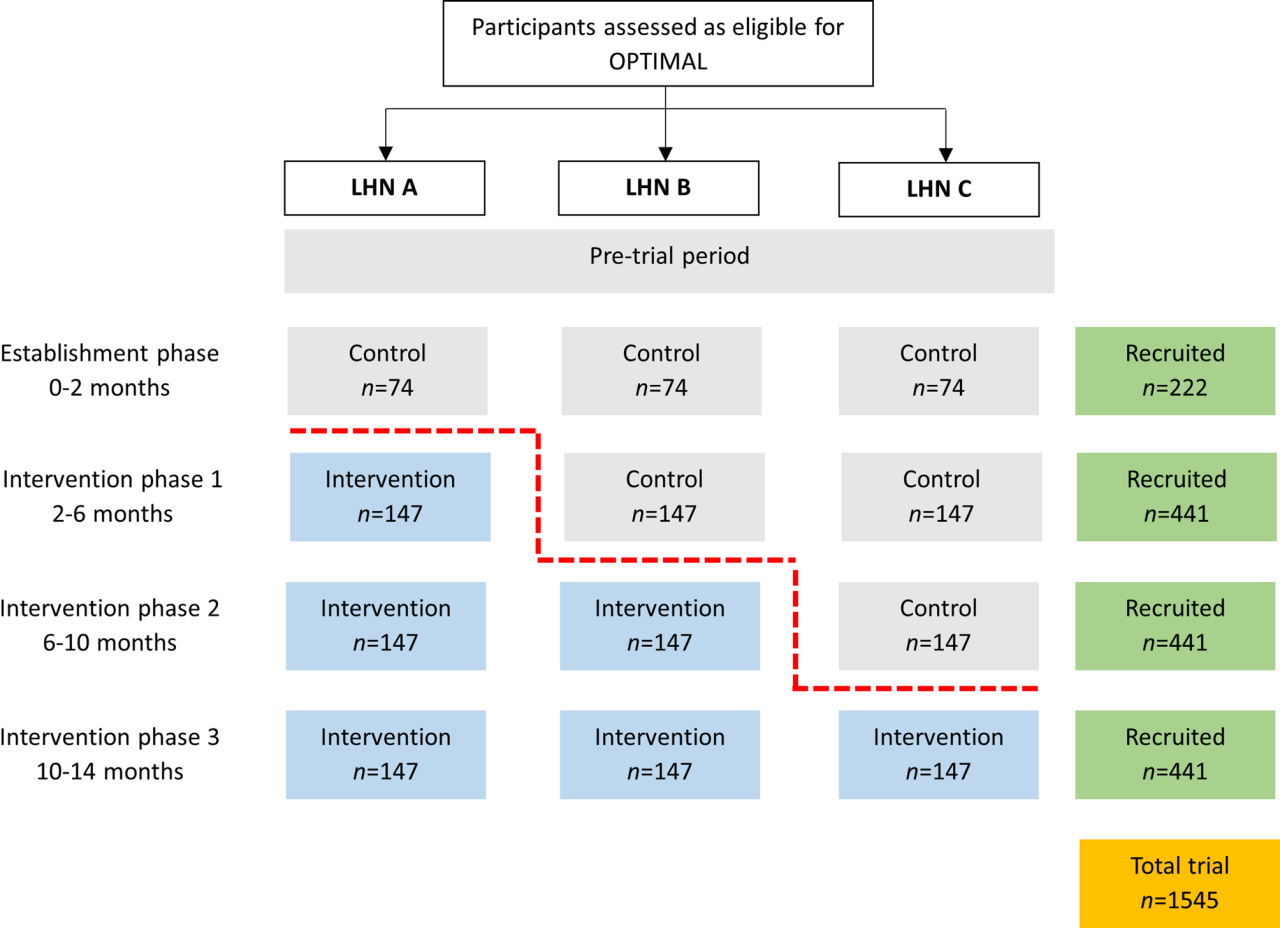
OPTIMAL study design

A total of 1545 participants are expected to be enrolled from the three LHNs over the 14 months (Fig. 3). Implementation will occur in four steps with 74 participants per LHN in the first step, and 147 participants per LHN per step in subsequent steps (Fig. 3). This sample size was calculated to ensure that the study has sufficient statistical power to detect a reduction from 20% to 10% in the proportion of patients readmitted within 90 days of discharge, with 80% power, a significance level of 5% and accounting for 10% attrition due to deaths or withdrawals.

### Recruitment

A waiver of the requirement for consent has been granted by the reviewing ethics committee as the intervention has no direct patient involvement, involves enhancements to patient clinical care, and uses routinely collected data from the EMR. The trial will be carried out across selected acute and sub-acute care wards in the three LHNs, selected to support achievement of required sample size. Data of all eligible patients from the selected hospital wards will be collected until the target sample size is reached. A poster will be displayed in participating wards to inform patients and families about the study. Should a patient contact the research team with objections to being included, they will be excluded from the study.

### Randomisation and blinding

The LHNs will be allocated to the intervention phase by simple randomization using a randomization table generated by Microsoft Excel. All LHN teams will be notified of their intervention commencement date prior to starting the trial, to allow adequate time for planning activities in the establishment phase and recruitment of the nurse facilitator. Blinding of the clinical teams carrying out the intervention is not possible due to the nature of the intervention.

### Data collection, management and analysis

ED presentations, hospital readmissions, deaths and days alive and in RAC will be identified from administrative data, which will be reviewed at 30 days and 90 days after entry into RAC. These outcomes will be identified from all EMR activated hospitals in South Australia and will exclude ED presentations and readmissions to private hospitals, as these are not captured.

Other variables required for adjustment of potential confounding factors during analysis of outcomes (such as demographic variables, clinical history and hospitalisation-related variables) will be extracted from the EMR. The data will be extracted and de-identified at the source, and the de-identified data will be stored electronically on a secure research server that is only accessible by the core research team.

Prior to analysis, the data will be checked and cleaned. Data will be analysed to check for any between group differences in potential confounding variables. Results will be reported using the CONSORT 2010 guidelines for randomised trials with the extension for stepped-wedge trials [38]. In addition to initial descriptive analysis, one-way analysis of variance (ANOVA) will be used to test group differences, and exploratory analysis will be conducted to compare participants of different demographics.

The proportion of patients discharged to residential aged care for the first time who present to ED and/or are readmitted and/or who died within 30 and 90 days will be analysed using binomial regression models with ED presentation, readmission or death (Yes/No) as a binary response variable, with the main covariate being whether the discharging ward is in control or intervention phase. Days alive and in RAC will be analysed for all patients using a generalised linear regression model. The models will adjust for potential patient related confounders including patient age, sex, and clinical history, and we will add a linear time covariate to account for any potential time trend over the period of data collection.

### Data collection for the process evaluation

The fidelity of implementation will be assessed through activity logs maintained by nurse facilitators in each LHN. The acceptability, adoption and feasibility will be assessed through qualitative interviews (both pre-implementation and post-implementation) with key decision makers from each LHN. The interview guides are based on the i-PARIHS framework, and participants will include a nurse health lead in each LHN, nurse unit managers from each LHN, doctors and nurse managers in selected wards, and a senior allied health member of multi-disciplinary teams. A trained researcher will conduct the qualitative interviews either face-to-face or on-line (via Microsoft Teams), depending on the participant’s preference. Interviews will be recorded (with participants’ permission) and transcribed. Transcripts will be de-identified by removing the participants’ names. The interviews will be supplemented by a short online survey administered to clinical managers and members of the clinical team in the selected sites. Survey questionnaire responses will be recorded using the Qualtrics platform and exported from Qualtrics to an Excel spreadsheet for analysis.

Thematic analysis of interview notes and transcripts from the pre-implementation and evaluation interviews will be carried out, supplemented by survey responses. Analysis will be iterative to identify emerging themes and then refined. The transcripts will be analysed by an experienced qualitative researcher using NVivo software to code qualitative data into categories then narrowed down into overarching themes, using the i-PARIHS as a conceptual framework. A selection of qualitative interviews will be analysed by a second member of the research team to check for agreement in coding. Any discrepancies will be discussed, and the coding frame clarified. Further details of the process evaluation protocol is given in a separate paper (Fernando RL, Crotty M, Inacio MC, Batta I, Bourke A, Forward J, Furst C, Whitehead C, Shaw S, Shepperd L, Harvey G: Implementing a bundle of interventions to support older adults transitioning from hospital to residential aged care: a protocol for the process evaluation of the OPTIMAL stepped wedge cluster randomised controlled trial, under review).

### Economic analysis

An economic analysis will incorporate intervention costs and costs associated with ED presentations and inpatient admissions. The main intervention cost will be the cost of employing a nurse to develop and facilitate the transition care model at each site. Costs will also be estimated for the time input of other staff towards the provision of implemented transition care models, informed by data collected for the process evaluation. Separate intervention costs will also be estimated at each site for the transition care provided to the low, moderate and high risk of readmission groups. Costs of ED presentations and inpatient admissions will be estimated using published cost data for Urgency Related Groups and Diagnostic Related Groups, respectively.

ED presentation and inpatient admission costs will be estimated for each participant, Site- and risk of readmission-specific intervention costs will be added to these costs for each intervention phase participant. Costs will be analysed for all patients using a generalised linear regression model, adjusting for potential patient related confounders and including a linear time covariate to account for potential time trends in costs. Cost-effectiveness will be estimated using bootstrapping to represent the joint uncertainty around the costs and outcomes.

### Data availability and dissemination

The final deidentified trial dataset will initially only be accessible to members of the core research team. This trial involves enhancement of care and uses routinely collected data; therefore, a data monitoring committee was not established. Data will be archived and retained for 15 years. Requests for access to the quantitative data for future research will be reviewed on a case-by-case basis by the principal investigator.

Results will be published in an international peer-reviewed journal, and any required data will be provided as a clean, de-identified data file. Substantive contributors to the trial will be recognised as part of the *OPTIMAL Project Research Collaboration Group*. Study findings will also be shared through consumer forums, stakeholder discussions and advocacy to policy and planning levels of government to accelerate translation and project influence at scale.

## Discussion

The OPTIMAL study will harness the available evidence on interventions to improve care transitions of older adults to provide valuable evidence on the transferability and effectiveness of introducing multicomponent and risk stratified enhanced care bundles, specific to older adults transitioning from hospital to RAC, for use in the Australian context. Improving the quality of transition care for this vulnerable group is expected to reduce unplanned ED presentations and hospital readmissions and improve patient outcomes. It also provides an opportunity to improve downstream workflows to address challenges with hospital access, with the additional benefit of reducing healthcare costs associated with readmissions.

The study uses the pragmatic stepped wedge design, allowing incremental and sequential roll out of the intervention across LHNs that mimics the logistical constraints of implementation in practice, while allowing for randomisation in the measurement of effectiveness [39]. This design enables the research team to focus attention and work closely with the LHN team during each establishment phase. The existing evidence supports the beneficial nature of the interventions, thus the unidirectional implementation design that covers all participating LHNs is also more ethically acceptable [40]. A drawback of the stepped wedge design is the confounding effect of time, which may reduce the precision of the study, but this will be accounted for in the analysis by introduction of a time covariate, and may also be mitigated by each LHN acting as its own control, which helps to increase the precision of the study [39].

The OPTIMAL intervention involves delivery of an evidence-based bundle of interventions to patients being discharged to RAC for the first time. Each LHN team will be responsible to select the bundle of interventions to be delivered at their sites, and considering the available resources, which will contribute to the acceptability and sustainability of implementation. The risk of contamination is minimised by randomising at the level of the LHN (cluster), thus the introduction of the data dashboard, employment of the nurse facilitator, and crossover to the intervention phase will be done at the level at the LHN. This design has the added advantage of allowing each LHN to be randomised to the intervention phase at its own time step, supporting the study to achieve optimal power [40].

As per the Medical Research Council guidance on theory-informed approaches to developing and evaluating complex interventions, implementation of the OPTIMAL intervention will be standardised in relation to the underlying process and key functions of the core components [35, 41]. Thus, the integrity of the intervention implementation will be defined based on function rather than composition [41], and trial implementation will focus on consistency at the level of mechanisms. The outcomes are expected to be achieved through the mechanisms of *simplifying* the care transition process, *verifying* essential steps, *connecting* providers, and *monitoring* the clinical status participants [24]. The OPTIMAL intervention logic model (given in the OPTIMAL process evaluation protocol paper (Fernando RL, Crotty M, Inacio MC, Batta I, Bourke A, Forward J, Furst C, Whitehead C, Shaw S, Shepperd L, Harvey G: Implementing a bundle of interventions to support older adults transitioning from hospital to residential aged care: a protocol for the process evaluation of the OPTIMAL stepped wedge cluster randomised controlled trial, under review)) describes the intended relationships between the determinants, components, implementation strategies, mechanisms and outcomes [42].

The trial will use routinely collected patient data extracted from the EMR which feeds into a data dashboard that identifies and risk stratifies eligible patients and allows extended follow up for evidence of the outcomes. This is advantageous in that it reduces the burden and cost of recruitment, risk stratification, data collection and outcome assessment [43], however it limits the outcomes that can be assessed and the potential confounders which can be accounted for in the analysis. Factors related to the type of RAC facility cannot be accounted for in the risk stratification and analysis. A major limitation in the use of secondary data for research is related to standardisation and completeness of data [43]. A drawback of this dependency on the EMR data for participant identification is the reliance on health professionals to accurately complete the relevant fields in the EMR. This was considered in the planning phase, and a modification to the EMR will be introduced to standardise data entry to identify eligible patients. In addition, health staff will be educated on the importance of accurate data entry to the EMR and provided with user guides for both the EMR modification and the data dashboard to minimise missing data and misclassification risks.

Care transitions interventions are known to act through a range of mechanisms, and the context of implementation will vary between the LHNs. The concurrent process evaluation is a methodological strength, providing an understanding of the underlying mechanisms and contextual factors affecting implementation and outcomes, and the transferability of findings. The i-PARIHS framework is ideal to understand the complex and dynamic nature of implementation with regards to the innovation, recipients and context (at the local, organisational and external health system level) of implementation, as well as the central role of a facilitator [33]. This provides an opportunity to identify enablers and barriers to implementation of such interventions, which will be useful when considering scaling up and implementation at other sites. Lessons can be drawn about whether, and in what configuration, the models of care should be expanded to other sites and can guide the reconfiguration of services for older patients with complex needs and possible changes to funding of health care programs. This is important to improve the quality of transition care for older persons, to secure better health outcomes for this vulnerable group.

## Supplementary Information

Supplementary Material 1. Components of the OPTIMAL intervention.

Supplementary Material 2. OPTIMAL SPIRIT Checklist.

## Data Availability

No datasets were generated or analysed during the current study.

## Abbreviations

RAC: Residential Aged Care
ED: Emergency Department
OPTIMAL: Optimising older People's Transition from acute care Into residential aged care through Multidisciplinary Assessment and Liaison
i-PARIHS: integrated Promoting Action on Research Implementation in Health Services
LHN: Local Health Network
NALHN: Northern Adelaide Local Health Network
CALHN: Central Adelaide Local Health Network
SALHN: Southern Adelaide Local Health Network
EMR: Electronic Medical Record
