# Supplementary material for: Optimising older People’s Transition from acute care Into residential aged care through Multidisciplinary Assessment and Liaison (OPTIMAL): protocol for a stepped wedge cluster randomised controlled trial with embedded process evaluation
Source: BMC Geriatr. 2025 Jul 28;25:550. doi: 10.1186/s12877-025-06187-y (PMC12302694; doi:10.1186/s12877-025-06187-y)
Supplement: Supplementary file 1 — Supplementary Material 1. Components of the OPTIMAL intervention. [file 12877_2025_6187_MOESM1_ESM.docx]

Supplementary File 1: Core components of the OPTIMAL intervention with permissible adaptations

| **OPTIMAL core component** | | **Permissible adaptations** |
| --- | --- | --- |
| 1 | Identification of older persons being discharged to residential aged care for the first time. | None |
| 2 | Risk stratification of eligible patients using data dashboard. | Number of risk categories to be decided by local team: either two or three levels of risk (low/high or low/medium/high). |
| 3 | Delivery of risk stratified bundle of evidence-based interventions to provide enhanced care to eligible patients. | Specific interventions and responsibility for delivery to be decided by LHN team based on the local context and may include a post discharge follow-up phone call from a nurse, medication reconciliation, access to a geriatric hotline and a post-discharge visit from a nurse.  Bundle of interventions should include at minimum   - a standard, same day discharge summary for all patients, and - case management by a registered nurse for those at high risk. |
| 4 | Implementation facilitation by a locally appointed nurse facilitator | Options for role sharing and distribution of 0.6 FTE.  Nurse facilitator can deliver interventions and/or coordinate delivery from other services/units. |
